# Entomo-virological investigation in urban forest fragments and intradomiciles during a dengue outbreak in Salinas, MG, Brazil

**DOI:** 10.1590/0074-02760250086

**Published:** 2026-01-26

**Authors:** Cirilo Henrique de Oliveira, Thaynara de Jesus Teixeira, Rudá Mahayana Cordeiro de Barros, Arlei Bispo de Araújo, Aline Tátila Ferreira, Danielle Costa Capistrano Chaves, Fabrício Souza Campos, Luiz Marcelo Ribeiro Tomé, Natalia Rocha Guimarães, Talita Émile Ribeiro Adelino, Felipe Campos de Melo Iani, Luiz Carlos Júnior Alcantara, Walter Santos de Araújo, Filipe Vieira Santos de Abreu

**Affiliations:** 1 Instituto Federal do Norte de Minas Gerais, Laboratório de Comportamento de Insetos, Salinas, MG, Brasil; 2 Universidade Estadual de Montes Claros, Programa de Pós-Graduação em Biodiversidade e Uso dos Recursos Naturais, Montes Claros, MG, Brasil; 3 Secretaria Municipal de Saúde de Salinas, Instituto Federal do Norte de Minas Gerais, Centro Colaborador de Entomologia, Laboratório de Comportamento de Insetos, Salinas, MG, Brasil; 4 Secretaria de Saúde do Estado de Minas Gerais, Coordenação Estadual de Vigilância de Arboviroses e Controle Vetorial, Belo Horizonte, MG, Brasil; 5 Universidade Federal do Rio Grande do Sul, Instituto de Ciências Básicas da Saúde, Porto Alegre, RS, Brasil; 6 Fundação Ezequiel Dias, Serviço de Virologia e Riquetsioses, Belo Horizonte, MG, Brasil; 7 Fundação Oswaldo Cruz-Fiocruz, Instituto René Rachou, Belo Horizonte, MG, Brasil; 8 Universidade Estadual de Montes Claros, Centro de Ciências Biológicas e da Saúde, Departamento de Biologia Geral, Laboratório de Interações Ecológicas e Biodiversidade, Montes Claros, MG, Brasil; 9 Fundação Oswaldo Cruz-Fiocruz, Instituto Oswaldo Cruz, Laboratório de Insetos Transmissores de Hematozoários, Rio de Janeiro, RJ, Brasil

**Keywords:** mosquitoes, DENV, forest fragments, spillover, arboviruses, Aedes, Culex

## Abstract

**BACKGROUND:**

Mosquitoes (Diptera: Culicidae) are among the most important disease vectors worldwide. Several species exhibit high levels of anthropophily and are frequently found in human dwellings and forest fragments near urban areas.

**OBJECTIVES:**

In this integrative study combining mosquito collection, viral detection, and ecological analyses, the assemblage of diurnal mosquitoes was investigated across three distinct environments - intradomiciles, and two distinct urban forest fragments (UFFs) - during a dengue outbreak in the city of Salinas, Minas Gerais, Brazil.

**METHODS:**

Sampled mosquitoes were tested for the presence of dengue, Zika, and chikungunya viruses through real-time quantitative polymerase chain reaction (RT-qPCR).

**FINDINGS:**

A total of 722 mosquitoes were collected, representing seven genera and 12 species. The most abundant species were *Culex quinquefasciatus* (270/722, 37.4%), *Aedes aegypti* (205/722, 28.4%), *Ae. albopictus* (112/722, 15.5%), and *Ae. scapularis* (110/722, 15.2%). Five of 81 pools tested positive for dengue virus serotype 1 (DENV-1) RNA, all belonging to *Ae. aegypti* species. Phylogenetic analyses of the nearly complete genome of DENV-1 revealed clustering with strains sampled in 2023 from São Paulo State. Mosquito richness and composition differed between environments (houses and urban forests), whereas abundance was similar across all environments.

**MAIN CONCLUSIONS:**

Important vector species were detected, including *Ae. aegypti*, *Ae. albopictus*, *Cx. quinquefasciatus*, *Ae. scapularis*, *Sabethes albiprivus*, and *Coquillettidia venezuelensis*, associated with the transmission of dengue, oropouche, mayaro, yellow fever, and Venezuelan equine encephalitis viruses. Entomological and virological investigations in urban and peri-urban environments are crucial, as these areas provide shelter and refuge for anthropophilic and opportunistic mosquito species. Our findings underscore a high potential for mosquito-borne disease spillover in these areas.

Arboviral diseases are a group of infectious illnesses caused by viruses transmitted by arthropods, primarily mosquitoes (Diptera: Culicidae). Dengue virus (DENV), chikungunya virus (CHIKV), Zika virus (ZIKV), and yellow fever virus (YFV) are notable examples of arboviruses that pose a significant public health challenge worldwide.^1^ The clinical manifestations of arbovirus infections range from mild febrile illness to severe neurological, arthritic, and haemorrhagic syndromes.^2^

In recent years, Brazil has experienced multiple outbreaks of arboviral diseases. the year 2024 has been marked by a record increase in cases of chikungunya, dengue, and Zika across the country compared to previous years (2021, 2022, and 2023). As of June 2024, the state of Minas Gerais reported 1,637,716 probable cases of DENV (935,899 cases and 733 confirmed deaths), 139,367 probable cases of CHIKV (105,664 cases and 75 confirmed deaths), and 271 probable cases of ZIKV (35 confirmed cases and no deaths).^4^

The species *Aedes aegypti* and *Ae. albopictus* are the main vectors of urban arboviruses such as DENV, ZIKV, and CHIKV.^5^ In Brazil, *Ae. aegypti* is a highly anthropophilic species that prefers urban areas, particularly within and around residences. In contrast, *Ae. albopictus* is more commonly associated with rural and peri-urban forested environments.^6^ ^,^ ^7^ ^,^ ^8^ Urban landscapes are heterogeneous mosaics, interspersing various types of land use and cover, including built areas and highly fragmented vegetation.^9^ Forest fragments within the urban environment can provide variable availability of breeding sites, facilitating the maintenance of diverse mosquito species,^10^ ^,^ ^11^ as well as vertebrate hosts for blood-feeding.^12^ This dynamic supports the survival and proliferation of anthropophilic and opportunistic/generalist sylvatic mosquitoes. Consequently, this landscape matrix, comprising green areas within or near urban zones, may heighten the risk of spillover of sylvatic viruses (*e.g.*, YFV, oropouche, and West Nile viruses) to humans and spillback of urban viruses such as DENV and CHIKV to wild hosts.^13^ ^,^ ^14^

Thus, given the need to prioritize more effective strategies for vector monitoring and control, it is essential to understand mosquito species diversity across different habitats and to assess virus infection rates. Accordingly, the objective of this study was to monitor the arboviruses DENV, CHIKV, and ZIKV during a dengue outbreak by analysing the adult mosquito fauna in urban areas and urban forest fragments (UFFs) of Salinas, Minas Gerais. This work represents the first entomo-virological investigation in northern Minas Gerais, that simultaneously integrates molecular arbovirus detection with ecological characterisation of mosquito assemblages in UFFs and intradomiciliary settings during an active dengue outbreak. We also report and phylogenomically analyse the first complete DENV-1 genome from an *Ae. aegypti* population in this region.

## MATERIALS AND METHODS

**Fig. 1:**
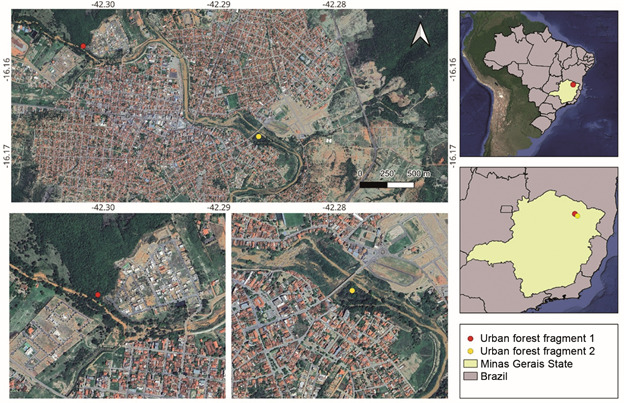
map showing the city of Salinas, where mosquito captures were conducted. Sampling sites in urban forest fragments (UFFs) are indicated by red and yellow dots. The figure was created using the free software QGIS 3.40.3.

*Study area* - This study was conducted in the city of Salinas (16º09’45.8′′ S; 042°17′54.2′′ W), located in northern Minas Gerais (Fig. 1). Salinas has a population of 40,178 inhabitants and a low municipal human development index (MHDI = 0.679).^15^ The study area lies in an ecotone between the Cerrado and Atlantic Forest biomes,^16^ with the Seasonal Deciduous Forest (commonly referred to as Dry Forest) as its main phytophysiognomy.^17^ The region has a semi-arid climate (Aw according to Köppen’s classification),^18^ characterised by two distinct seasons: a prolonged dry season from March to October and a short rainy season from November to February.

*Mosquito collection* - Collections were conducted between February and June 2024, across three distinct environments ― intradomiciles, and two distinct UFFs ― during a dengue outbreak. Methods and protocols were previously approved by Brazilian Ministry of the Environment (SISBIO nº 75826-4). Urban mosquito captures were scheduled based on residents’ availability, and intradomiciliary mosquitoes were collected and processed as previously described.^19^ Briefly, household visits were scheduled according to residents’ availability and conducted by a municipal vector surveillance agent and an entomologist equipped with battery-powered Nasci aspirators, oral aspirators, and entomological cages. Sampling involved thorough inspection of all rooms, with special attention to hidden niches such as under furniture and behind cabinets. A total of 36 houses were sampled, with an average collection time of 50 minutes per house, totaling 30 h of collection effort. Peri-urban mosquitoes were captured using the protected human attraction method^20^ with entomological nets and Castro aspirators conducted by two collectors simultaneously in two UFFs. The first site (UFF 1) is a riparian urban forest of the Ribeirão stream (Fig. 1), located near a newly developed neighbourhood. This site comprises a small forest fragment connected to a larger fragment, and adjacent to pastures (Fig. 1). The second site (UFF 2) is a riparian urban forest along the Salinas River, situated in the city centre and surrounded by an urban matrix (Fig. 1). A total of 20 h of sampling was conducted in each UFF, amounting to 40 h of total sampling effort.

After capture, mosquitoes were sorted by genus and sex and transferred to field cages using oral aspirators. The cages were then sealed, labelled, and transported to the Insect Behaviour Laboratory at the Federal Institute of Northern Minas Gerais. Mosquitoes were kept alive for three days, as previously described.^19^ Subsequently, they were euthanised by freezing at -20ºC, transferred to cryovials, and stored in liquid nitrogen (-196ºC) until further processing.

*Taxonomic identification and molecular diagnosis of captured mosquitoes* - Mosquitoes were transferred from liquid nitrogen and subjected to identification and taxonomic confirmation on a cold table (-20ºC) under a stereoscopic microscope, using dichotomous keys.^21^ ^,^ ^22^ Non-engorged mosquito bodies were pooled (up to 10 individuals) by species and sex. Each mosquito’s head was carefully separated from its body under a stereomicroscope, using a sterile scalpel blade dedicated to that specimen to prevent cross-contamination. Mosquito processing and RNA extraction were performed as previously described.^19^ Briefly, individual heads and pools of up to 10 bodies from the same species and sex were placed in enriched L-15 medium (Thermo Fisher Scientific Cat. No. 11415064; enriched with 20% foetal bovine serum (FBS), 0.5% non-essential amino acids, 1% penicillin, 0.1% gentamicin, and 0.1% fungizone). Tissues were homogenised with beads in a beadbeater for 30 s at 7500 rpm, followed by centrifugation at 12,000 rpm for 8 min at 4ºC. A 140 µL aliquot of the resulting supernatant was used for RNA extraction with the QIAamp Viral RNA Mini Kit (Qiagen, Cat. No. 52906), according to the manufacturer’s instructions. RNA from body pools was tested for DENV, ZIKV, and CHIKV by real-time quantitative polymerase chain reaction (RT-qPCR) using the Bioclin ZDC Multiplex One-Step kit (Cat. No. G021-3), with 9 µL of total RNA as input, following the manufacturer’s protocol. For DENV-positive pools, RNA from each mosquito head was individually tested to confirm viral dissemination. DENV-positive pools were also subjected to a specific one-step RT-qPCR assay to identify the serotype (DENV-1 to DENV-4) using a previously described protocol, with 5 µL of total RNA as input.^23^ All samples were tested in duplicate. Samples were considered positive when the cycle threshold (Ct) was below 40.

*DENV genome sequencing and phylogenomic analyses* - The RNA extracted from the DENV-1 positive pool (X-737) was subjected to cDNA synthesis and PCR amplification following a sequencing protocol based on the multiplex PCR-tiling amplicon approach.^24^ The resulting amplicons were then purified using 1× AMPure XP Beads (Beckman Coulter, Brea, CA, USA, Cat. No. A63880) and quantified with a Qubit 3.0 fluorometer (Thermo Fisher Scientific, Waltham, MA, USA) using the Qubit™ dsDNA HS Assay Kit (Thermo Fisher Scientific, Cat. No. Q32851).

The sequencing library was prepared using the Native Barcoding Kit 96 V14 (SQK-NBD114.96) from Oxford Nanopore. Sequencing was performed on a MinION device (Oxford Nanopore) for 24 h, using the Flow Cell R10.4.1 (FLO-MIN114) and the MinKNOW software (v24.02.16) with basecalling disabled. Basecalling and demultiplexing of the libraries were conducted using Guppy software (v6.5.7).

*Bioinformatics a*nalysis - The genome of *Orthoflavivirus denguei* serotype 1 (DENV-1) was assembled using the reference-based assembly approach, implemented in a custom pipeline. This pipeline integrates the following tools: NanoPlot (v1.43.0, github.com/wdecoster/NanoPlot) for quality assessment, Chopper (v0.8.0) for adapter and low-quality base removal, Minimap2 (v2.28) for alignment, Samtools (v1.20) for alignment file manipulation, Bcftools (v1.20) for variant calling, iVar (v1.4.2) for consensus genome generation, and Pilon (v1.24) for correction and refinement of the final assemblies. The reference sequence was obtained from the NCBI under the accession number NC_001477.1, corresponding to DENV-1. The genome quality assessment and genotyping were performed using Nextclade (v3.10.0). The newly generated DENV-1 genome sequence has been deposited in GISAID under accession number EPI_ISL_19725409.

For the phylogenetic analysis, the assembled genome of sample X-737 was initially aligned with 2,527 DENV-1 genomes obtained from the GISAID database, with collection dates ranging from 2022-11-02 to 2024-12-17 [Supplementary data (Table)]. The alignment was performed using the MAFFT software and was manually inspected in AliView. Subsequently, a preliminary phylogenetic analysis was conducted using FastTree (v2.1.11). Based on the generated phylogenetic tree, the most representative clade containing the X-737 genome was identified. From this clade, a subset of sequences was selected, resulting in a set of 619 DENV-1 genomes, which were aligned with the X-737 genome and inspected as previously described. The final alignment file was used to infer the most suitable evolutionary model and to perform the maximum likelihood (ML) phylogenetic analysis using IQ-TREE2 (v2.3.6). The statistical robustness of the tree topology was assessed using 1000 bootstrap replicates.

*Ecological analysis* - Generalised linear models (GLMs) were constructed to test the effect of the environment ― houses, UFF 1, and UFF 2 (explanatory variables) ― on mosquito richness and abundance (response variables). To assess mosquito abundance, we employed GLMs with a negative binomial distribution due to overdispersion in the data. For species richness, we adopted GLMs with Poisson distribution. All models were subjected to residual analysis using the testDispersion function from the “DHARMa” package^25^ to assess the adequacy of the error distribution.^26^ Subsequently, all models were analysed using analysis of variance (ANOVA), with significance assessed using the χ² test for the abundance model and the F test for the richness model.^26^ To determine whether the areas differed significantly from each other, contrast analysis was also performed. Non-Metric Multidimensional Scaling (nMDS) based on the Bray-Curtis similarity index was used to compare species composition between urban, and two forest fragments environments. A non-parametric permutation procedure (ANOSIM) with 999 permutations, also based on the Bray-Curtis similarity index, was then applied to test the significance of the groups formed in the nMDS. All statistical analyses were performed using R software.^27^

## RESULTS

*Species collected and infection rates* - A total of 722 mosquito specimens were collected, representing 12 taxonomic units, with 10 taxa identified at the species level. Of the 395 mosquitoes collected in the urban environment (intradomiciles), all belonged to the subfamily Culicinae, tribes Aedini, and Culinici, comprising only two genera: *Culex* and *Aedes*. The most abundant species was *Culex quinquefasciatus* (n = 203), followed by *Ae. aegypti* (n = 191) and *Ae. scapularis* (n = 1). In the UFFs (UFF 1 and 2), the 327 specimens were distributed across seven genera and 12 taxa. The most abundant species were: *Ae. albopictus* (n = 112; 34.3%), *Ae. scapularis* (n = 109; 33.3%), and *Cx. quinquefasciatus* (n = 67; 20.5%) [Table I and Supplementary data (Table)]. *Ae. aegypti* and *Wyeomyia* sp. were exclusively detected at UFF1, while *Mansonia humeralis*, *Ae. serratus*, *Coquillettidia venezuelensis*, *Psorophora albipes*, and *Cx.* (*Melanoconion*) spp. were restricted to UFF2 [Supplementary data (Table)].

**TABLE I:** Species, number, relative abundance, and infection rate of mosquitoes sampled

Species	Number of mosquitoes		Total	Relative abundance (%)	No. tested - Pools tested (DENV positive pools)	MIR
	House (%)	UFF (%)				
*Culex quinquefasciatus* Say, 1823	203 (51.4)	67 (20.0)	270	37.4	260 - 25 (0)	0
*Aedes aegypti* (Linnaeus, 1762)	191 (48.3)	14 (4.3)	205	28.4	179 - 35 (5)	27.9
*Aedes albopictus* (Skuse, 1894)	0	112 (34.3)	112	15.5	64 - 7 (0)	0
*Aedes scapularis* (Rondani, 1848)	1 (0.3)	109 (33.3)	110	15.2	104 - 11 (0)	0
*Psorophora ferox* (Humboldt, 1819)	0	9 (2.8)	9	1.2	5 - 2 (0)	0
*Culex* (*Mel.*) spp. Theobald, 1903	0	7 (2.1)	7	1.0	0 - 0 (0)	0
*Mansonia humeralis* Dyar & Knab 1916	0	3 (0.9)	2	0.4	2 - 1 (0)	0
*Sabethes albiprivus* Theobald, 1901	0	2 (0.6)	2	0.3	2 - 0 (0)	0
*Aedes serratus* (Theobald, 1901)	0	1 (0.3)	1	0.1	0 - 0 (0)	0
*Coquillettidia venezuelensis* (Thobald, 1912)	0	1 (0.3)	1	0.1	0 - 0 (0)	0
*Psorophora albipes* (Theobald, 1907)	0	1 (0.3)	1	0.1	0 - 0 (0)	0
*Wyeomyia* sp. Theobald, 1907	0	1 (0.3)	1	0.1	0 - 0 (0)	0
Total	395 (54.3)	327 (45.7)	722	100.0	614 - 81 (5)	-

DENV: dengue virus; MIR: minimum infection rate = number of positive pools / number of same species adults analysed × 1000; UFF: urban forest fragment (the sum of UFF 1 and 2).

The mosquito bodies were grouped into 81 pools. All pools tested negative for CHIKV and ZIKV. Notably, five *Ae. aegypti* pools (comprising 28 mosquitoes) tested positive for DENV-1, as did six individual heads, with CT values ranging from 26.4 to 28.4 (Table II).

**TABLE II:** Description of dengue virus serotype 1 (DENV-1)-positive pools

Cod. pool	Species	No. of individuals	Sex	Cycle threshold (Ct)	Positive heads
X723	*Aedes aegypti*	7	F	27.7	ITc417; ITc421
X733	*Ae. aegypti*	6	F	27.6	ITc467
X737	*Ae. aegypti*	5	F	26.4	ITc487
X757	*Ae. aegypti*	5	F	26.5	ITc554
X760	*Ae. aegypti*	5	F	28.4	ITc567

**Fig. 2:**
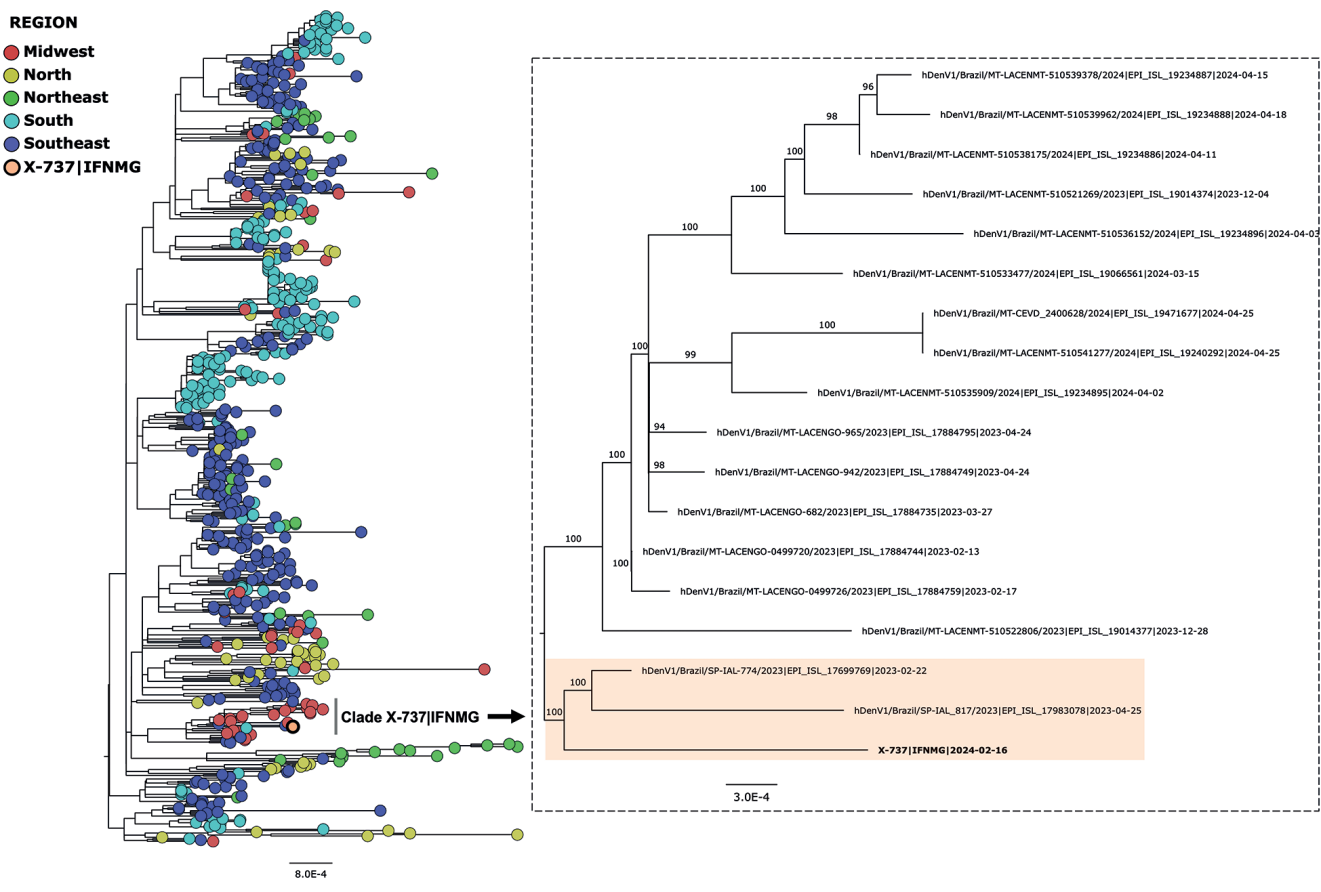
maximum likelihood (ML) phylogenetic tree generated in IQ-TREE using the TIM2+F+I+R5 evolutionary model with 1000 bootstrap replicates. The tree was constructed using a dataset of 619 dengue virus serotype 1 (DENV-1) genomes from the GISAID database, along with the genome generated in this study from sample X-737. In the phylogenetic tree on the left, the genome generated in this study is highlighted, showing the clade in which it clustered. On the right, an expanded view of the subclade containing the X-737 genome is displayed, with the genomes highlighted in orange.

*DENV genome sequencing and phylogenomic analyses* - Sequencing of sample X-737 generated an *O. denguei* genome with 93.60% coverage and an average depth of 2,150X (26,334 reads mapped to the reference genome). Genome quality assessment, conducted using the online tool Nextclade, confirmed the high quality of the genome and classified it as dengue virus serotype 1 (DENV-1), genotype V. Maximum likelihood (ML) phylogenetic analysis revealed that the newly generated genome clustered with genomes from the Midwest and Southeast regions. Specifically, it was more closely related to two genomes from São Paulo (Southeast region), collected in February and April 2023 (Fig. 2).

**Fig. 3:**
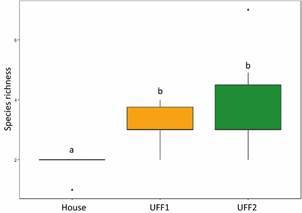
comparison of mosquito species richness among houses, urban forest fragment 1 (UFF1) and UFF2. Different letters indicate significant differences between the bars.

*Ecological analyses* - Species richness differed significantly (F = 3.626; P = 0.026) among the treatments (environments), with houses exhibiting lower species richness compared to forest environments (Fig. 3). In contrast, mosquito abundance showed no significant difference between environments (Chisq = 34.759; p = 0.507).

**Fig. 4:**
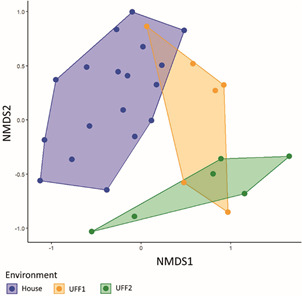
non-metric multidimensional scaling (nMDS) showing the ordination of mosquito species composition across house, urban forest fragment 1 (UFF1) and UFF2 environments.

Significant differences in mosquito assemblage composition were observed across the environment types (ANOSIM: R = 0.510; P = 0.001), with the most pronounced difference occurring between houses and UFFs. This is visually evident in the NMDS diagram, which shows a distinct grouping of samples by environment type (Fig. 4).

## DISCUSSION

The urban and peri-urban mosquito fauna in Salinas, northern Minas Gerais, was investigated during the largest dengue outbreak in recent years. We identify *Ae. aegypti* mosquitoes, as the primary vector of DENV-1, with no evidence of other species implicated in the transmission of this virus. Most entomo-virological surveillance has focused on highly urbanised settings, particularly households, and targeted only the main vectors, *Ae. aegypti* and *Ae. albopictus*.^28^ ^,^ ^29^ ^,^ ^30^ ^,^ ^31^ In contrast, studies in UFFs have mainly described mosquito fauna without virological screening^32^ ^,^ ^33^ ^,^ ^34^ or conducted entomo-virological monitoring during periods without active viral circulation.^35^ Our study addresses this gap by jointly assessing mosquito assemblages and arbovirus circulation in both households and UFFs, emphasising their relevance for detecting potential spillover/spillback events and for strengthening early warning systems.

Dengue is currently the most significant mosquito-borne viral disease globally.^36^ ^,^ ^37^ In 2024, between epidemiological weeks 1 and 34, approximately 6,500,000 probable cases of dengue were reported in Brazil, with 5,244 confirmed deaths attributed to the disease.^38^ DENV consists of four antigenically distinct serotypes, capable of triggering explosive outbreaks of varying magnitudes.^39^ ^,^ ^40^ Both DENV-1 and DENV-2 were circulating throughout the state of Minas Gerais in 2024, causing human cases.^4^ ^,^ ^41^ Among the infected mosquitoes, we identify only the DENV-1 serotype, which predominated in this outbreak, including in the study region.^4^ The clade formed by the DENV-1 genome sequenced from Salinas (MG) and two genomes from São Paulo (SP), collected in February and April 2023, possibly reflects the high rate of human migration from northern Minas Gerais to São Paulo in search of work, as well as the absence of DENV-1 genomes from local human cases, due to limited infrastructure.

The high frequency of *Cx. quinquefasciatus* and *Ae. aegypti* indoors is consistent with the primarily urban and anthropophilic habitat of these vectors.^21^ However, despite the higher abundance of *Cx. quinquefasciatus*, only *Ae. aegypti* was found naturally infected with DENV-1, including viral detection in the head, indicating virus dissemination throughout the mosquito’s tissues. Notably, this is the first DENV-1 genome sequenced from an *Ae. aegypti* population in Minas Gerais. The minimum infection rate (MIR) for *Ae. aegypti* observed in this study is higher than those previously reported in the literature (MIR = 1.47 for adult *Ae. albopictus* collected in spring 2014 in São Paulo, Brazil, and 3.37 and 16.2 for immature and adult *Ae. aegypti*, respectively, collected in Rio Grande do Norte, Brazil, between 2011 and 2014).^42^ ^,^ ^43^ The high indoor abundance of mosquitoes, combined with the MIR (27.9) observed in *Ae. aegypti*, underscores the role of this species in maintaining and transmitting DENV in Brazilian urban environments.^42^

Although the primary dengue vector in the Americas is the urban mosquito *Ae. aegypti*, *Ae. albopictus* exhibits high vector competence and has been found naturally infected with DENV (including DENV-1) in Brazil.^42^ ^,^ ^44^ Albeit the presence of *Ae. albopictus* in ovitraps in the peridomestic areas of Salinas has been documented,^45^ this species was not found inside the sampled domiciles. In Brazil, *Ae. albopictus* typically exploits forest edges in transitional areas (ecotones) between forests and urban landscapes, positioning it as a potential bridge vector for arboviruses between these environments.^46^ This behaviour aligns with our findings, as *Ae. albopictus* was the most abundant species in the sampled UFFs. Interestingly, one specimen of *Ae. scapularis* was found inside one sampled domicile. While the presence of *Ae. scapularis* indoors may be incidental, this species has previously been documented indoors during a CHIKV outbreak^47^ and has been found naturally infected with YFV.^48^ ^,^ ^49^ Both *Ae. albopictus* and *Ae. scapularis* are potential arbovirus vectors, exhibiting ecological versatility, adaption to anthropogenic environments, and eclectic blood-feeding behaviour.^22^ ^,^ ^50^ ^,^ ^51^ ^,^ ^52^ ^,^ ^53^ Therefore, these species warrant attention, as they can feed on a wide variety of vertebrates found in urban green areas, in addition to humans, potentially acting as bridge vectors for zoonotic transmission.^12^ ^,^ ^21^

Urban green spaces - while essential for ecosystem services and human well-being - also constitute pivotal ecological niches that influence arbovirus dynamics. In forest edges and UFFs, the co-occurrence of vector species underscores such areas’ potential as bridge zones linking sylvatic and peri-urban transmission cycles.^46^ ^,^ ^54^ In the Brazilian Amazon, the detection of ZIKV in *Ae. aegypti* and *Ae. albopictus* collected within UFFs further highlights how such green spaces may facilitate pathogen circulation.^31^ Urban greening efforts must be paralleled by vigilant, ecologically informed arbovirus monitoring to effectively mitigate human health risks. Here, the mosquito fauna detected in the small UFFs suggests that these urban green spaces, even when heavily modified by human activity, provide favourable environments for the persistence of medically important mosquito species. Beyond *Ae. scapularis* e *Ae. albopictus*, other potential vectors identified include *Ps. ferox* and *Sabethes albiprivus*, which have been previously found naturally infected with YFV^55^ ^,^ ^56^ and *Cq. venezuelensis*, a vector of oropouche and Venezuelan equine viruses.^57^ ^,^ ^58^ Casual collections included species highly adapted to urban environments, such as *Cx. quinquefasciatus* and *Ae. aegypti*. The persistence and abundance of these vectors in urban green areas underscore their opportunistic behaviour and reflect the ongoing process of anthropisation.

We observe differences in species richness and composition, while species abundance was similar across the three environments (intradomicile, UFF1, and UFF2). Previous studies have also shown that mosquito fauna can vary between indoor and outdoor environments associated with households.^59^ ^,^ ^60^ ^,^ ^61^ ^,^ ^62^ Although it is beyond the scope of this study, differences in species richness and composition are likely influenced by the association of mosquitoes with landscape types.^63^ Heterogeneous landscapes support greater mosquito diversity compared to simplified landscapes, as they provide a higher availability of breeding sites^11^ and a greater abundance of food resources for mosquitoes.^12^ ^,^ ^64^ The houses and UFFs shared three species, among which *Cx. quinquefasciatus* was the most abundant. The high abundance of *Cx. quinquefasciatus* in urban environments is directly related to its ability to breed in polluted water bodies rich in organic matter.^65^ Such water collections were observed in the streets as well as in an open sewage channel near the UFF1 sampling site. Finally, the exclusive presence of five sylvatic mosquito species in UFF2 highlights the ability of small forest fragments, even when surrounded by urban landscapes, to sustain diverse vector populations.

The absence of DENV, CHIKV, and ZIKV detection in mosquitoes collected from the UFFs, even during recent epidemics in the city of Salinas,^19^ suggests that these viruses were not actively circulating in these environments. Sylvatic cycles of DENV and ZIKV persist in Asia and Africa, where both viruses continue to spill-over to humans and are occasionally translocated to new continents.^66^ ^,^ ^67^ ^,^ ^68^ Furthermore, reports in the literature describe natural infection of the sylvatic mosquito *Haemagogus leucocelaenus* in Brazil,^69^ raising concerns about the potential establishment of a sylvatic dengue cycle. Therefore, continuous entomo-virological surveillance is essential to monitor the risk of sylvatic transmission cycles of DENV and ZIKV becoming established in the Americas.^70^ ^,^ ^71^ Although the risk of spillover or spillback involves a complex interplay of factors,^72^ the presence of known vector species identified in this study highlights a concerning issue for public health authorities.

Finally, it is important to acknowledge some limitations of our study. Mosquito collections were conducted during an active dengue outbreak and over a restricted temporal window (February to June 2024). These conditions may have influenced the observed species composition, infection rates, and viral circulation patterns, thereby limiting the generalisability of our findings to other time periods or non-outbreak contexts.

*In conclusion* - Our study underscores the critical importance of investigating mosquito fauna in conjunction with virological analysis in urban and peri-urban forested areas, which are often excluded from municipal control and surveillance programs. Despite the limitations of our study, such as the short sampling period, our findings contribute to understanding the role of mosquito fauna in the epidemiology of DENV and provide valuable insights into the dynamics of mosquito assemblages in urban green areas. Collectively, these data can inform the development of strategies to assess the receptivity of different areas to arboviruses and guide the design of preventive measures aimed at managing the transition between urban and forest environments, with a focus on species that may serve as bridge vectors. Integrating entomological and virological surveillance across domiciliary and green areas strengthens early warning systems by detecting arboviruses in field-caught vectors and enabling timely control measures. Furthermore, the molecular monitoring approach can be incorporated into routine entomological programs to enhance preparedness and support evidence-based outbreak prevention strategies.

## SUPPLEMENTARY MATERIALS

Supplementary material

## Data Availability

The authors declare that all data supporting the findings of this study are available within the paper. The database used in this work for phylogenetic analysis was created using DENV-1 genomic sequences deposited in the GISAID EpiArbo database (GISAID Identifier: EPI_SET_250212gv; doi: 10.55876/gis8.250212gv). We sincerely thank all the laboratories and researchers who generated and shared these sequences, enabling advances in genomic surveillance and research. For details about the contributors, including accession numbers, virus names, collection dates, originating and submitting laboratories, and author lists, please visit 10.55876/gis8.250212gv. The DENV-1 genome generated in this study has been deposited in the GISAID EpiArbo database under the following Accession ID: EPI_ISL_19725409.
