# Supplementary material for: Entomo-virological investigation in urban forest fragments and intradomiciles during a dengue outbreak in Salinas, MG, Brazil
Source: Mem Inst Oswaldo Cruz. 2026 Jan 26;120:e250086. doi: 10.1590/0074-02760250086 (PMC12834460; doi:10.1590/0074-02760250086)
Supplement: Supplementary material [file 1678-8060-mioc-120-e250086-s.pdf]

TABLE

Mosquito species composition, abundance, and richness recorded in each urban forest fragment (UFF)

Taxa	UFF 1	UFF 2	Total
	No. (Relative abundance %)	No. (Relative abundance %)	
Aedes albopictus (Skuse, 1894)	79 (44.6)	33 (22.0)	112
Aedes scapularis (Rondani, 1848)	16 (9.0)	93 (62.0)	109
Culex quinquefasciatus Say, 1823	65 (36.7)	2 (1.3)	67
Aedes aegypti (Linnaeus, 1762)	14 (7.9)	0 (0)	14
Psorophora ferox (Von Humboldt, 1819)	1 (0.6)	8 (5.3)	9
Culex (melanoconion) spp. Theobald, 1903	0 (0)	7 (4.7)	7
Mansonia humeralis Dyar & Knab 1916	0 (0)	3 (2.0)	3
Sabethes albiprivus Theobald, 1903	1 (0.6)	1 (0.7)	2
Aedes serratus (Theobald, 1901)	0 (0)	1 (0.7)	1
Coquillettidia venezuelensis (Theobald, 1912)	0 (0)	1 (0.7)	1
Psorophora albipes (Theobald, 1907)	0 (0)	1 (0.7)	1
Wyeomyia spp. Theobald, 1901	1 (0.6)	0 (0)	1
Abundance	177	150	327
Richness	7	10	12
